# Under pressure to exercise: a cross-sectional study of characteristics and predictors of compulsive exercise in early adolescents

**DOI:** 10.1186/s40337-022-00686-8

**Published:** 2022-11-05

**Authors:** S. Bratland-Sanda, S. K. Schmidt, M. S. Reinboth, K. A. Vrabel

**Affiliations:** 1 Department of Sport, Physical Education and Outdoor Studies, University of South-Eastern Norway, Gullbringvegen 36, 3800 Bø, Telemark, Norway; 2 Research Institute, Modum Bad Psychiatric Centre, Vikersund, Norway; 3 Institute of Psychology, University of Oslo, Oslo, Norway

**Keywords:** Physical fitness, Mental health, Health promotion, Quality of life, Sports, Behavioral medicine

## Abstract

**Background:**

To investigate the frequency of compulsive exercise among early adolescents, and determine the associated impact of sex, physical activity level, exercise habits, motivational regulation, dieting behaviour and health-related quality of life (HRQoL) on compulsive exercise.

**Methods:**

Cross-sectional design with 8th grade adolescents (n = 572, mean ± SD age 13.9 ± 0.3 yrs). Outcome assessment was compulsive exercise (Compulsive Exercise Test, CET). Total CET score ≥ 15 was defined as clinical CET score. Further assessment included exercise motivation (Behavioural Regulation of Exercise Questionnaire—2), HRQoL (KIDSCREEN 27), accelerometer-assessed physical activity and Andersen test for cardiorespiratory fitness. Exercise obsession was defined as clinical CET score and < 60 min/day with moderate-to-vigorous objectively assessed physical activity.

**Results:**

Small sex differences were found for CET total score. Seven percent of the adolescents were classified with clinical CET score, and four percent with exercise obsession. Adolescents with clinical CET score had higher body mass index, more weight loss attempts, and lower physical fitness compared to adolescents with non-clinical CET score. Being a boy, higher scores on introjected motivational regulation and HRQOL subscale parent relation and autonomy, use of exercise monitoring tool, and number of weight loss attempt the past 12 months explained 39% of the total CET score variance. Physical activity level did not predict compulsive exercise.

**Conclusions:**

Compulsive exercise in early adolescents was predicted by exercise motivation, exercise habit, and dieting, but not physical activity level. This implicates a distinction of obsessive cognitions about physical activity from performed physical activity in adolescents, and that such cognitions must be addressed in future initiatives that aim to improve adolescents’ general physical activity level, health, and wellbeing.

*Trial registration* ClinicalTrials.gov: NCT03906851.

**Plain English summary:**

Although there is a huge concern about adolescents being insufficiently physically active, there are also adolescents who struggle with issues of compulsive exercise. The issues of compulsive exercise have been rarely studied in adolescents. We therefore aimed to describe compulsive exercise and factors that were associated with and could explain presence of compulsive exercise. A total of 572 8th graders (age 13.9 ± 0.3 yrs) responded to this study. We found that the score on compulsive exercise was higher in boys than in girls, and that adolescents with high score on compulsive exercise had higher body mass index, more weight loss attempts, and lower physical fitness compared to adolescents with low score on compulsive exercise. Also, we found that exercise obsessions, i.e., thinking of exercise without actually exercising, was present in four percent of the respondents. Being a boy, attempting weight loss, exercising to avoid shame/guilt, and exercising for the perceived value of exercise predicted compulsive exercise. Awareness of the compulsive exercise and exercise obsessions is important in public health initiatives that aim to increase adolescents’ physical activity level.

## Introduction

Low levels of physical activity (PA) is a major public health concern, leading the World Health Organization (WHO) to initiate a global action plan with the aim of reducing low levels of PA by 20% within 2030 [1]. Adolescence is the life phase with the steepest decline in PA levels, and this decline often continues into adulthood [2]. Despite this knowledge, adolescence as a life phase is understudied compared to other life phases [3]. Many strategies and initiatives have already been executed to promote more PA among adolescents with various level of success [4]. Unfortunately, in trying to move the young generation, the issue of PA and exercise becomes more complex as some adolescents develop an unhealthy, obsessive, and compulsive relationship to exercise [5, 6].

Compulsive exercise can be viewed in light of the DSM-5 criteria for obsessive–compulsive disorder and is defined as exercise obsessions and compulsive actions driven by avoidance of the discomfort of exercise deprivation [7]. It is detected as the most common symptom of eating disorders in youth [8], viewed as a step towards use of other pathogenic weight-control methods [9], and acknowledged as a major burden for those affected [10]. The few studies examining compulsive exercise in the general adolescent population report inconsistency regarding prevalence differences between sexes [5, 11–13]. Compulsive exercise is predicted by dysfunctional emotion regulation [11]. Other identified predictors are socio-cultural risk factors such as feedback from family and peers about muscularity in boys and perceived thinness pressure from media in girls [5, 14, 15]. Plateau et al. [16] found higher levels of both compulsive exercise and disordered eating behaviour in adult users versus nonusers of exercise monitoring tools. Whether the use of such monitoring tools is linked to compulsive exercise among adolescents is unknown. Further, dimensions of compulsive exercise are associated with impaired quality of life in both clinical eating disorder and general populations [17].

Although the level of compulsive exercise is linked to athlete populations [18] and high PA levels [19, 20], a recent study showed that exercise obsessions without actual compulsive exercise behaviour is detected in 20% of hospitalized adult patients with longstanding eating disorders [19]. Poobalan et al. [21] found that intentions to exercise is not necessarily associated with actual exercise behaviour, this impacts our understanding of cognitions about exercise in the absence of actual performed PA and exercise. In general treatment of obsessive–compulsive disorders, exposure and response prevention consists of exposing whatever elicits the anxiety or discomfort in different ways, and then learn how to resist any compulsion to stop the anxiety instead of employing obvious or subtle avoidance [22]. Given the complexity of compulsive exercise, the focus on one side is to stop the compulsion and the irresistibly internal urge to perform dysfunctional exercise behaviour. On the other side, it is important to encourage continuance with functional exercise behaviour. This complexity requires more understanding and knowledge.

Motivation behind exercise has been described specifically as the key antecedents of compulsive exercise [23]. As motivation determines the initiation, maintenance, and completion of relevant behaviours, analysing adolescents’ quality of motivation may be an important key to further understand compulsive exercise among this group. Using self-determination theory (SDT) [24] as a framework, we can examine the degree to which people engage in their actions with a complete feeling of choice. Moreover, SDT suggests that motivation toward any given behaviour can be either extrinsically, intrinsically or amotivated. These categorizations of motivation represent different degrees of internalization of external values and goals, and therefore differ in the degree to which they are self-determined or autonomous [24]. Amotivation refers to an absence of either extrinsic or intrinsic motivation, no perceived value of the activity, nor a belief that engaging in the activity will result in any personally meaningful outcomes. Extrinsic regulation is comprised of four different types of regulation: external regulation (i.e., perceived pressure to engage in the activity or to obtain some external incentive); introjected regulation (i.e., activity performed to avoid feelings of guilt, shame and anxiety); identified regulation (i.e., more self-determined type of motivation where the benefits and outcomes of the activity are valued as greater than the potential unpleasantness); and integrated regulation (i.e., the behaviour connected to the activity is constructed in congruence with the other values and needs that make up one’s personality) [24]. It is, according to SDT, only when individuals perform a behaviour because of the inherent fun and enjoyment in the activity, that the behaviour is fully intrinsically motivated. A comprehensive review by Standage and Ryan [25] showed that self-determined motivational regulations are related to more adaptive outcomes compared to controlling regulations and amotivation. To date, only a limited number of studies have examined the relationship between the motivational regulations established by SDT and forms of compulsive exercise [13, 26, 27]. Overall, most of these studies show that higher scores on introjected regulation, more specifically avoidance of shame and guilt, seems to be the strongest predictor of unhealthy forms of exercise (i.e., compulsive exercise and exercise dependence). However, they also show that more self-determined forms of motivation (e.g., integrated and identified regulations) are correlated with compulsive exercise, thus not fully confirming the predictions by SDT.

The overall aim of this study was to examine compulsive exercise in a Norwegian sample of early adolescents. We wanted to explore potential sex differences, differences in adolescents with and without clinical levels of compulsive exercise, and determine how PA level, exercise habits and motivation, dieting behaviour, and health-related quality of life (HRQoL) relate to compulsive exercise.

## Materials and methods

In this article, we report on cross-sectional data from the follow-up of the study “Active and Healthy Kids in Telemark”. Assessment of compulsive exercise, which is the primary outcome variable for this current publication, was only obtained at this time point of the study. We report on inclusion and exclusion criteria, all included assessments, and statistical analyses used. More information about this study has been published previously [28]. Anonymized data and analysis codes are available from main author upon request.

### Sample

The sample consisted of adolescent pupils in their first year of secondary school. Inclusion criterion was enrolment to 8th grade (i.e., age 13–14 years) at one of the 15 public secondary schools in the six municipalities selected by Telemark County in the school year 2017/2018. Exclusion criteria were absence from school on data collection day, language barriers that could affect the self-report, and for physical fitness testing and PA assessment the presence of illness and/or injury at the time of data collection was reason for exclusion.

### Design

**Fig. 1:**
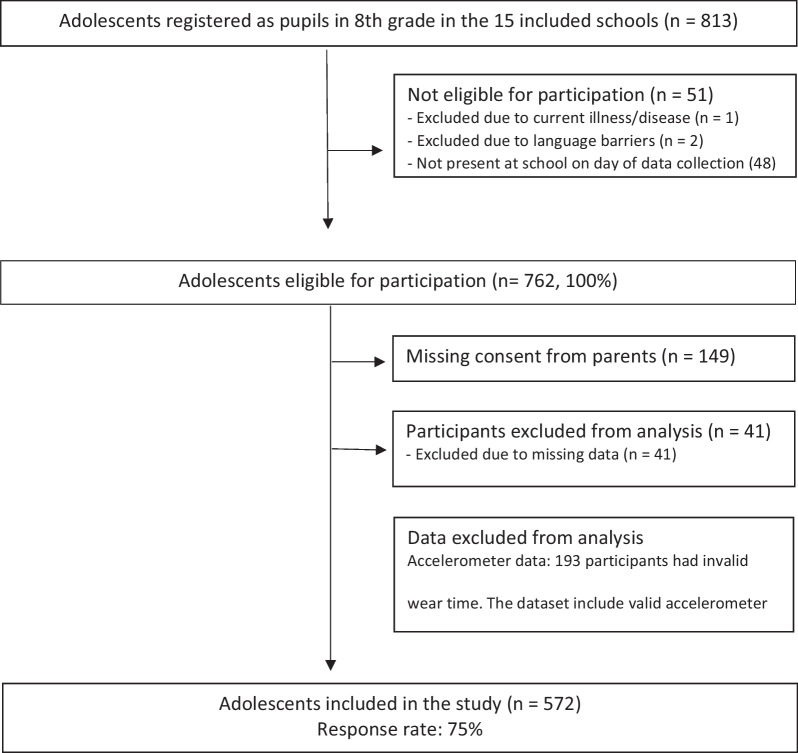
Flow chart of the study

Data were obtained during one school day in April and May 2018. Pupils absent from school on the day of data collection were therefore unable to participate (Fig. 1). Prior to baseline and follow-up data collection, written information about the study was distributed to the pupils and their parents, and parents gave written consent to the pupils’ participation in the study. The study was conducted in accordance with the Helsinki Declaration, was evaluated by the Regional Committee for Medical and Health Research Ethics (ID 2017/387), approved by the Norwegian Data Protection Services (ID 54327) and registered in ClinicalTrials.gov (NCT03906851).

### Instruments

#### Accelerometer-assessed PA level

Level of PA was objectively measured by triaxial accelerometer (ActiGraph GT3X+, LLC, Pensacola, FL, USA) worn for 4 consecutive days (2 weekdays and 2 weekend days). The accelerometer was worn on the right hip for the entire day, except during water-based activities or while sleeping. Accelerometers were initialized to start recording at 06:00 on the day after they were distributed. Epoch length was set to 10-s intervals. The criterion for a valid day was set as a wear time of 480 min/day between 06:00 and 24:00. A total of ≥ 2 days (weekdays and/or weekend days) was the criteria for a valid measurement. All sequences of ≥ 20 min of consecutive zero counts from each subject’s recording were excluded and defined as non-wear time, as this indicates periods in which participants did not wear the accelerometer. We used ActiLife software (ActiGraph, LLC, Pensacola, FL, USA) to initialize the monitors and to download the accelerometer files. The outcomes for total PA were counts per minute (cpm) from the accelerometer’s vertical axis (cpm axis 1). Sedentary time was defined as all activities less than 100 cpm, a threshold that corresponds with sitting, reclining or lying down [29]. Evenson’s criteria [29] were used to define cut-offs for moderate-to-vigorous PA. We analysed all accelerometer data using ActiLife software (ActiGraph, LLC, Pensacola, FL, USA).

#### Survey data on exercise habits

Questions on exercise habits were developed by the researchers, resulting in the following categorical variables: (1) Weekly participation in various types of exercises and/or sports (i.e., did they participate in any form of exercise: yes/no), (2) Arena for participation (i.e., did they participate in organized sport:yes/no, and/or exercise at fitness training center: yes/no), and (3) Use of exercise monitoring tools (i.e., did they use any type of exercise monitoring tool: yes/no, and if yes, did they use mobile phone applications (yes/no) and/or heart rate monitors (yes/no)).

#### Compulsive Exercise Test (CET)

The CET is a 24-item, self-report questionnaire validated in both general and clinical adolescent and adult populations [30, 31] and translated and validated in Norwegian by Vrabel and Bratland-Sanda [19]. The items are answered on a 6-point Likert scale ranging from 0 (never true) to 5 (always true). The items are divided into five subscales (avoidance and rule-driven behaviour, weight control exercise, mood improvement, lack of exercise enjoyment, and exercise rigidity). The CET total score is calculated by summing each mean subscale score. The Cronbach’s alpha for CET total score in this sample was 0.88, with subscale alpha ranging from 0.67 to 0.90. A CET total score of 15 or higher has been identified as a clinical cut off [31], and this cut off score was used in this sample. To examine frequency of exercise obsessions, i.e., preoccupation with exercise without actually exercising as described in Bratland-Sanda et al. [32], all respondents with CET total score ≥ 15 and < 60 min/day with moderate-to-vigorous PA were classified with exercise obsession. The cut off for daily moderate-to-vigorous PA corresponds to the WHO recommendations and guidelines for daily PA among adolescents [33].

#### Behavioural Regulation of Exercise Questionnaire (BREQ-2)

The Norwegian version of the Behavioural Regulation in Exercise Questionnaire-2 [34]. The BREQ-2 comprised 19 items and five factors: amotivation (e.g., “I don’t see the point in exercising”), extrinsic regulation (e.g., “I exercise because other people say I should”), introjected (e.g., “I feel guilty when I don’t exercise”), identified (e.g., “It’s important to me to exercise regularly”) and intrinsic motivation (e.g., “I exercise because it’s fun”). Participants scored responses on a Likert-type scale ranging from 0 to 4, where 0 = “not true for me” and 4 = “very true for me”. Cronbach’s alpha range 0.78-0.89 for the various regulations.

#### HRQoL (KIDSCREEN-27)

The KIDSCREEN-27, which is a shorter version of the original KIDSCREEN-52, assesses physical, psychological, social and behavioural components of HRQoL [35]. It consists of the five domains “Physical health”, “Psychological health and well-being”, “Social support and peers”, “Parent relations and autonomy” and “School environment”. The Cronbach’s alpha ranged from 0.83 to 0.88 for the various domains in this sample.

#### Weight regulation

The respondents were asked about number of attempts of weight gain and weight loss the past 12 months.

#### Physical fitness

The 10-min intermittent running field measurement Andersen test was used to determine physical fitness. The test is performed by running back and forth in a sport hall with 20 m width in periods of 15 s work and 15 s rest [36]. The outcome was expressed as distance covered in meters. This test has been found reliable and valid for adolescents on a group level [37].

#### Anthropometric measures

The adolescents wore light clothing and no shoes for body height and body weight measurements. Both height and body weight were measured in the school nurse’s office at each school. Height measurements were collected using a wall-mounted standardized stadiometer and were measured to the nearest 0.1 cm. Body weight was measured to the nearest 0.1 kg using an ADE electronic weight (ADE, Hamburg, Germany), and electronic scale weights. Body mass index (BMI) was calculated as weight in kilograms divided by height in squared meters (kg m^−2^).

### Statistical analyses

IBM SPSS 26.0 was used for the analyses of the data. To account for potential bias in the missing data, we conducted a Little’s MCAR test. This test showed a *p* value of 0.09, which indicates that the data were missing completely at random. The CET total score and subscale score variables were checked for normal distribution using skewness (variables range from 0.08 to 1.14) and kurtosis (variables range from 0.03 to 1.40), all variables were within the suggested range for normal univariate distribution [38, 39]. Descriptive data were presented as mean *(SD)* and n (%), whereas independent t-tests and chi square were used to examine differences between sexes and between respondents above and below CET cut off. Effect sizes were calculated using Cohen’s *d* for continuous variables and *r* for categorical variables. The effect sizes were classified as small (*d* = 0.20 and *r* = 0.10), medium (*d* = 0.50 and *r* = 0.30) and large (*d* = 0.80 and *r* = 0.50). Pearson’s correlation coefficient was used to examine associations between CET total and subscale score and the following variables: BMI, physical fitness, motivational regulation of exercise, PA level, exercise arena, use of exercise monitoring tools, weight regulation, and HRQoL. A multiple, hierarchical linear regression was conducted with CET total score as dependent variable and independent variables chosen from previous theoretical findings such as motivational regulation and physical activity level [13, 16, 18–20], and from the significant correlations (i.e., BMI, motivational regulation for exercise, exercise arena, use of exercise monitoring tools, weight regulation, and HRQoL). The variables were included in the analysis in the following blocks and order: sex (block 1), PA level, use of exercise monitoring tools, and arena for exercise (block 2), BMI (block 3), motivational regulation of exercise (block 4), HRQoL (block 5), and weight regulation (block 6) as independent variables. A VIF score above 2.5 indicated collinearity as suggested by Johnston et al. [40]. Significance level was 0.05.

## Results

### Descriptive data

Of the 572 respondents, 288 were boys (50.3%) and 284 (49.7%) were girls. Most of the respondents reported weekly participation in exercise and/or sports (531 [92.8%]), and 199 (34.6%) of the respondents reported use of monitoring tools during exercise (Table 1). Regarding sex differences, a higher frequency of girls was active in organized sports compared to boys (*p* < 0.001), and a higher frequency of boys exercised at fitness training centres compared to girls (*p* < 0.05). Table 1 also showed sex differences in attempts of weight loss (*p* < 0.001) and weight gain (*p* < 0.001) the past 12 months, BMI (*p* < 0.05) and scores on BREQ-2 intrinsic motivation (*p* < 0.05).

**Table 1:** Participants’ characteristics and measured by sex: boys (n = 288) and girls (n = 284)

Participants’ characteristics and measures	Boys	Girls	Diff	ES^a^
Continuous variables	mean (SD)	mean (SD)	t	d
Age, years	13.9 (0.3)	13.9 (0.3)	− 1.1	0.00
BMI, kg/m^2^	20.0 (3.4)	20.7 (3.3)	− 2.1*	0.21
PA level^b^, cpm	473.9 (217.8)	499.0 (263.6)	− 0.9	0.10
BREQ amotivation	0.2 (0.6)	0.2 (0.6)	0.4	0.00
BREQ external regulation	0.4 (0.6)	0.4 (0.7)	0.5	0.00
BREQ introjected regulation	1.1 (1.1)	1.2 (1.1)	0.3	0.09
BREQ identified regulation	2.1 (1.7)	2.2 (1.1)	1.6	0.07
BREQ intrinsic motivation	2.5 (1.2)	2.7 (1.2)	2.0*	0.17
CET total	10.0 (4.0)	9.3 (3.4)	2.0*	0.19
CET avoidance and rule-driven behaviour	1.5 (1.2)	1.3 (1.1)	2.1*	0.17
CET weight control	1.9 (1.1)	1.8 (1.2)	1.8	0.09
CET mood improvement	2.7 (1.3)	2.6 (1.2)	1.3	0.08
CET lack of enjoyment	1.2 (1.1)	1.2 (1.2)	0.9	0.00
CET rigidity	2.7 (1.3)	2.6 (1.3)	2.1	0.08

Categorical variables	N (%)	N (%)	χ^2^(1)	R
Weekly participation in exercise and/or sports	264 (92)	267 (94)	1.2	0.04
Exercise arena: organized sports	196 (68)	230 (81)	12.6***	0.15
Exercise arena: fitness training centre	51 (18)	31 (11)	5.4*	0.10
Use of exercise monitoring tools	92 (32)	107 (38)	2.1	0.06
Weight gain attempts past 12 months	44 (15)	19 (7)	10.8***	0.14
Weight loss attempts past 12 months	39 (14)	80 (28)	18.6***	0.18
Meeting PA recommendations^b^	59 (38)	61 (36)	0.2	0.02
Above CET cut off score (CET ≥ 15)	26 (9)	16 (6)	2.4	0.06

*BMI* body mass index (kg/m^2^). *PA* physical activity. *Cpm* counts per minute. *BREQ* Behavioural Regulation of Exercise Questionnaire. *CET* Compulsive Exercise Test

*p < 0.05. **p < 0.01. ***p < 0.001

^a^Effect size: Cohen’s *d* (continuous variables) and *r* coefficient (categorical variables). ^b^n = 379

### Compulsive exercise

Boys reported a higher mean CET total score and CET subscale avoidance score compared to girls (*p* < 0.05, Table 1). Seven percent of the respondents showed clinical CET score, with no difference in prevalence among boys and girls (Table 2). The adolescents with clinical CET score showed higher BMI (*p* < 0.01), lower cardiorespiratory fitness (*p* < 0.05), more frequent use of exercise monitoring tools (*p* < 0.001), exercising in fitness training centres (*p* < 0.05), and weight loss attempts (*p* < 0.001), and higher scores on amotivation (*p* < 0.05) and all extrinsic motivational regulations for exercise (*p* < 0.01 and *p* < 0.001) compared to adolescents with non-clinical CET score (Table 2). Of the 317 respondents with both complete CET score and valid accelerometer assessment, 11 (3.5%) showed both clinical CET score and insufficient PA level according to the WHO recommendations for adolescents [33], indicating exercise obsession. The adolescents with indications of exercise obsessions were five boys and six girls, due to the inadequate power we did not perform analysis of differences between the adolescents with clinical CET score, adolescents with exercise obsessions, and the adolescents with non-clinical CET scores.

**Table 2:** CET total score below (CET < 15) and above (CET ≥ 15) cut off points by participants characteristics and measures

Participants’ characteristics and measures	CET < 15	CET ≥ 15	Diff	ES^a^
Continuous variables: mean (SD)	*(n* = *530)*	*(n* = *42)*	*t*	*d*
Age*, years*	13.9 (0.3)	13.9 (0.3)	0.1	0.00
BREQ amotivation	0.2 (0.5)	0.4 (0.9)	2.3*	0.23
BREQ external regulation	0.4 (0.6)	0.9 (0.9)	4.8***	0.65
BREQ introjected regulation	1.1 (1.0)	2.1 (1.3)	6.0***	0.86
BREQ identified regulation	2.1 (1.1)	2.7 (1.3)	3.1**	0.50
BREQ intrinsic motivation	2.6 (1.2)	2.6 (1.3)	0.4	0.00
HRQoL Physical Health	46.3 (10.6)	48.9 (12.0)	1.5	0.23
HRQoL psychological health and wellbeing	49.8 (10.4)	47.0 (14.5)	1.6	0.22
HRQoL parent relations and autonomy	54.7 (11.8)	57.4 (11.8)	1.4	0.23
HRQoL social support and peers	49.9 (10.9)	54.3 (13.0)	2.5*	0.37
HRQOL school environment	50.8 (11.3)	52.8 (14.3)	1.1	0.16

	*(n* = *499)*	*(n* = *42)*		
BMI, kg/m^2^	20.3 (3.3)	21.9 (4.2)	3.1**	0.42

	*(n* = *459)*	*(n* = *39)*		
Physical fitness, meters	1007.9 (108.8)	971.2 (130.1)	2.0*	0.31

	*(n* = *297)*	*(n* = *20)*		
PA level, cpm	841.7 (320.5)	840.8 (195.1)	0.01	0.00

Categorical variables: n (%)			χ^2^ (1)	
Sex: boy/girl	262(91)/268(94)	26(9)/16(6)	2.4	0.06
Exercise arena: organized sports	494 (93)	37 (88)	1.5	0.05
Exercise arena: fitness training centre	71 (13)	11 (26)	5.2*	0.10
Use of exercise monitoring tools	174 (33)	25 (60)	12.2***	0.15
Weight loss attempts past 12 months	96 (18)	23 (55)	31.7***	0.24
Weight gain attempts past 12 months	58 (11)	5 (12)	0.04	0.01
Meet PA recommendations^b^	105 (35)	9 (45)	0.8	0.05

*BMI* body mass index (kg/m^2^). *PA* physical activity. *Cpm* counts per minute. *BREQ* Behavioural Regulation of Exercise Questionnaire. *CET* Compulsive Exercise Test

*p < 0.05. **p < 0.01. ***p < 0.001

^a^Effect size: Cohen’s *d* (continuous variables) and *r* coefficient (categorical variables)

### Correlation and prediction of CET score

CET total and subscale scores were associated with all motivational regulations (Table 3). The strongest, positive correlation was found between introjected regulation and the subscale “avoidance” (*r* = 0.58, *p* < 0.001). The strongest, negative correlation was found between intrinsic motivation and the subscale “lack of enjoyment” (*r* =  − 0.67, *p* < 0.001). CET total score was positively associated with participation in organized sports, use of exercise monitoring tools, dieting behaviour and the HRQoL domains physical health and school environment, but not associated with PA level (Table 3).

**Table 3:** Correlations of potential predictors with CET total score and CET subscale total scores

	CET total	CET avoidance and rule-driven behaviour	CET weight control	CET mood improvement	CET lack of enjoyment	CET rigidity
BMI, kg/m^2^	0.11*	0.08	0.26***	− 0.04	0.07	− 0.02
Physical fitness, metres	0.05	0.14*	− 0.14**	0.19**	− 0.28***	0.21***
BREQ Amotivation	− 0.02	− 0.03	0.02	− 0.17***	0.38***	− 0.20***
BREQ external regulation	0.24***	0.20***	0.27***	0.07	0.18***	0.06
BREQ introjected regulation	0.49***	0.58***	0.31***	0.43***	− 0.20***	0.36***
BREQ identified regulation	0.39***	0.43***	0.09*	0.53***	− 0.47***	0.53***
BREQ intrinsic motivation	0.21***	0.26***	− 0.09*	0.53***	− 0.67***	0.50***
PA level, cpm	0.04	0.07	− 0.09	0.17***	− 0.15**	0.11*
Exercise arena: organized sports	0.13**	0.13**	− 0.10*	0.24***	− 0.28***	0.30***
Exercise arena: fitness training centre	0.05	0.10*	0.10*	0.02	− 0.03	− 0.02
Use of exercise monitoring tools	0.18***	0.22***	0.12**	0.14**	0.001	0.06
Weight loss attempts past 12 months	0.28***	0.16***	0.49***	0.04	0.17***	0.04
Weight gain attempts past 12 months	0.01	0.03	− 0.03	0.02	− 0.002	0.01
HRQOL physical health	0.18***	0.20***	0.17***	0.39***	− 0.35***	0.41***
HRQOL psychological health and wellbeing	− 0.06	− 0.10	− 0.28***	0.18***	− 0.24***	0.20***
HRQOL parent relations and Autonomy	0.05	− 0.03	− 0.13**	− 0.23***	− 0.21***	0.24***
HRQOL social support and Peers	0.11	0.08	− 0.01	0.22***	− 0.16***	0.18***
HRQOL school environment	0.09*	0.05	− 0.13**	0.32***	− 0.32***	0.30***

*BMI* body mass index. *PA* physical activity. *BREQ* Behavioural Regulation of Exercise Questionnaire. *CET* Compulsive Exercise Test

*p < 0.05. **p < 0.01. ***p < 0.001

A hierarchical regression analysis with total CET score as dependent variable showed that gender (boy), use of exercise monitoring tools, introjected regulation, the HRQoL dimension of Parent relation and autonomy, and number of weight loss attempts the past 12 months predicted 39% of the variance of the CET score (Table 4). Two variables in the regression analysis, i.e., identified regulation and intrinsic motivation had VIF score above 2.5, indicating considerable collinearity.

**Table 4:** Predictors of compulsive exercise in adolescents

Independent variable	Model 1	Model 2	Model 3	Model 4	Model 5	Model 6
*Block 1*						
Sex	− 0.13*	− 0.14*	− 0.15*	− 0.12*	− 0.13*	− 0.16**
*Block 2*						
PA level, cpm		− 0.02	− 0.01	0.02	0.02	0.03
Use of exercise monitoring tools		0.21***	0.21***	0.15**	0.16**	0.15**
Exercise arena: organized sports		0.13*	0.13*	0.002	− 0.01	0.01
Exercise arena: fitness training centre		0.04	0.03	− 0.003	− 0.01	0.005
*Block 3*						
BMI, kg/m^2^			0.07	0.07	0.10	0.02
*Block 4*						
BREQ amotivation				0.06	0.06	0.07
BREQ external regulation				− 0.02	− 0.01	− 0.03
BREQ introjected regulation				0.44***	0.49***	0.46***
BREQ identified regulation				0.15	0.10	0.13
BREQ intrinsic motivation				− 0.09	− 0.14	− 0.13
*Block 5*						
HRQOL physical health					0.06	0.07
HRQOL psychological health and wellbeing					− 0.09	− 0.02
HRQOL parent relations and autonomy					0.14*	0.14*
HRQOL social support and peers					0.10	0.05
HRQOL school environment					0.07	0.04
*Block 6*						
Weight loss attempts past 12 months						0.22***
Weight gain attempts past 12 months						− 0.004
Adjusted R^2^	0.01	0.06	0.06	0.28	0.31	0.34

Use of standardized regression coefficients (β-value) from hierarchical linear regression analysis

Dependent variable: Total Compulsive Exercise Test score. *BMI* body mass index. *PA* physical activity. *BREQ* Behavioural Regulation of Exercise Questionnaire

*p < 0.05. **p < 0.01. ***p < 0.001

## Discussion

The aim of this study was to examine compulsive exercise in Norwegian early adolescents, and determine the associated impact of sex, PA level, exercise habits, motivational regulation for exercise, dieting behaviour and HRQoL on compulsive exercise.

The CET total scores in both boys and girls are comparable to the findings from the adolescent sample in two previous studies conducted in the UK [14, 15], but higher than in another UK study from the same research group [18]. Further, a higher total CET score in boys compared to girls, although the difference was small, was consistent with previous findings from French and US samples [12, 13]. The reason for this sex difference is believed to be related to a higher frequency of sport participation, weight lifting, and the use of exercise as a method to regulate weight and appearance among boys compared to girls [13]. However, our findings are somewhat contradictory to the latter hypothesis as we did not find any sex difference on the CET subscale weight control.

In our study, seven percent of the sample was defined with clinical CET score. In clinical samples from females with eating disorders, the frequency of clinical CET score has been found to be 52–54% [19, 31]. To our knowledge, we are the first to identify the construct of exercise obsessions in an adolescent sample. Compared to the 20% defined with exercise obsessions in the adult clinical sample in Vrabel and Bratland-Sanda [19], we argue that the high percentage found in our general adolescent sample calls for greater attention to adolescents’ thoughts and attitudes towards exercise in screening of adolescents’ exercise behaviour in Norwegian as well as other Western societies. The compulsive exercise beliefs may be seen independent from the associated behaviour as actual PA level was similar. However, physical characteristics (i.e., BMI and physical fitness), motivational regulation of exercise, and dieting behaviour differed between adolescents with clinical and non-clinical CET score. The small to moderate differences in BMI, physical fitness, weight loss attempts, and use of exercise monitoring tools, and the moderate to large differences in external, introjected and identified regulation for exercise found among adolescents with clinical compared to non-clinical CET score, implies that high scores on compulsive exercise in general adolescent populations is linked to maladaptive thoughts about exercise, and dieting behaviour. The higher frequency of using fitness training centres as an exercise arena has also been linked to more external regulation and less self-determined exercise motivation compared to organized sports as an exercise arena for adolescents [41]. This is in accordance with previous findings of exercise addiction prevalence in fitness training centres attendees versus organized sport participants [42]. Compared to Goodwin et al. [18] who found higher CET scores in sport versus non-sport participants, our findings might seem a bit surprising. Unfortunately, the study by Goodwin et al. [18] did not examine other arenas for exercise participation than competitive sports, and thus potential associations between body dissatisfaction and compulsive exercise in other exercise arenas in that study have not been reported. Overall, in the current study, it seems that the adolescents with clinical CET scores are preoccupied with exercise, losing weight, and perceive high levels of both internal and external pressure to exercise. However, they seem to lack the actual adaptive and healthy exercise behaviour. Some studies have found that effortful inhibition of eating and appetitive behaviours (i.e., dieting) can have negative cognitive effects in the sense that it leads to more obsessive thoughts about food and eating in adults [43]. It may be that, for this group, preoccupation with exercise is characterized by a driven urge to exercise, without the perceived inability to actually stop exercising. In our opinion, this calls for attention to exercise obsessions without compulsive exercise actions as an important part of the compulsive exercise construct. For exercise obsessions in adolescents, we applied the WHO recommendations of 60-min with daily moderate-to-vigorous PA [33] as a cut off for low levels of PA. This includes all forms of PA, i.e., school-based PA, active transportation, leisure-time PA, and structured exercise and sport participation. This can be viewed as a strict cut off which might be insufficiently specific and thus include “false positive”. However, it must be emphasized that different criteria can be considered for screening of the general population versus categorization of exercise obsession in e.g., clinical samples from various populations with eating disorders. It is also in our opinion important to acknowledge the vast amount of research and evidence-base behind the recommendations of a minimum of one-hour daily PA for health in this age group, and thus we argue that it is important not to pathologize PA amounts below this recommendation in the general population.

The highly significant predictors for compulsive exercise determined by the regression analysis, i.e., sex, use of exercise monitoring tools, introjected regulation, and weight loss attempts, are in accordance with findings from Ganson et al. [44], Plateau et al. [16] and Symons Downs et al. [13]. These findings are not surprising as compulsive exercise is a well-known approach to regulation of body weight and appearance in both clinical and non-clinical populations [20, 44, 45]. The adolescents’ use of monitoring tools for exercise can be viewed as one of several warning signs for compulsive exercise, and is therefore something parents, teachers and sport coaches must be aware of.

Interestingly, the adolescents’ level of objectively assessed physical activity was neither associated with nor did it predict the CET score. Based on previous findings in clinical populations, the construct of exercise as an eating disorder symptom is more linked to compulsivity than an excessive amount [46]. Although Symons Downs et al. [13] found more time reported spent in leisure-time exercise as predictor of compulsive exercise, this result was based on self-reported PA and exercise which has been found less valid compared to objective assessment of PA and exercise [47]. Our finding therefore adds to this knowledge in a general adolescent population.

All but one blocks used in the hierarchical multiple linear regression model provided significant changes to the model, with the greatest improvement in adjusted R^2^ from 6% (Model 3) to 28% (Model 4). Only the adding of BMI (Model 3) was not significant. The improvement of the model when adding the motivational regulation of exercise (Model 4) is in accordance with previous studies on the importance of these variables [13, 48]. One form of extrinsic motivation, i.e., controlled (introjected) motivational regulation of exercise, stood out as a core predictor for compulsive exercise. This is in accordance with findings from Symons Downs et al. [13], who found integrated regulation and introjected regulation to be important determinants of primary exercise dependence symptoms. Further, Thøgersen-Ntoumani and Ntoumanis [48] found introjected regulation of exercise more closely linked to both adaptive and maladaptive behaviour in adult exercisers. Introjected regulation of exercise motivation refers to behaviour performed to avoid feelings of guilt, shame, and anxiety, and hence covers the core of the compulsive exercise construct. Thus, it is not surprising that introjected regulation showed a moderate, positive correlation with CET avoidance and rule-driven behaviour. According to SDT, intrinsic motivation is all about the enjoyment of the behaviour, and the negative, strong correlation between intrinsic motivation for exercise and the CET subscale “lack of enjoyment” was expected. The strong, positive correlation of identified regulation with the CET subscale “rigidity” was also expected as the identified regulation refers to behaviour performed as a sense of obligation. Thus, perceived benefits outweigh the perceived disadvantages, regardless of perceived enjoyment of the activity.

Strengths of this study include a large sample of adolescents, high response rate, use of standardized and validated instruments, and the use of objective assessment of PA and physical fitness. Further, we believe it is a strength that we have a broad approach to exercise behaviour and thus can capture the complexity to a greater extent than previous studies. The cross-sectional design is a weakness together with the limited data on dieting behaviour. The study is also limited by the lack of data on disordered eating and eating disorders as this is important information when interpreting findings on dieting and compulsive exercise, and the huge difference in sample size between the groups with clinical and non-clinical CET score warrants caution when interpreting the results. Further, the lack of data on the adolescents that were absent from the school on the day of data collection limited our possibilities to examine potential response bias in the sample. As this study was conducted in Norway and similar studies with similar findings are from Western countries, this current study might therefore be most relevant for addressing compulsive exercise in Western cultures.

There is a need for longitudinal studies on how compulsive exercise evolves during childhood and adolescence, and whether it is linked to other mental health issues than eating disorders. Also, there is a need for separation of obsessive cognitions about exercise from actual performed exercise in adolescents, and knowledge about how such cognitions might serve as a great barrier towards healthy and joyful exercise participation. This warrants greater awareness about compulsive exercise in future initiatives and policy making aimed at increasing the general PA level in adolescents. There is a need also for studies with larger and more equal-sized samples that have adequate power to analyse potential differences in PA, physical fitness, dieting, disordered eating, eating disorders, and motivational regulation of exercise among adolescents classified with exercise obsession, compared to the adolescents with and without compulsive exercise. Further, we argue that compulsive exercise is a better marker than performed exercise in screening of unhealthy and maladaptive behaviour related to dieting and exercise. Practitioners such as physical education teachers, sport coaches, and clinical exercise physiologists can use the knowledge derived from our study, together with previous knowledge about the importance of creating autonomy- and need-supporting environment to improve intrinsic motivation and thus healthy adherence to exercise [49].

## Conclusion

Compulsive exercise scores were higher in boys compared to girls, although the differences were small. Seven percent were classified with clinical CET scores and four percent were classified with exercise obsessions. Differences in adolescents with and without clinical CET scores were identified, these differences were mostly small to moderate except for the large difference in introjected motivational regulation for exercise. Compulsive exercise in adolescents was predicted by sex, use of exercise monitoring tools, exercise motivational regulations, and weight loss attempts, but not PA level. The findings implicate separation of obsessive cognitions about PA from actual PA level in adolescents, and that such cognitions must be addressed in future PA promoting initiatives.

## Data Availability

The datasets generated and analysed during the current study are not publicly available due to GDPR and restrictions to the ethical approval about such availability. Anonymized data and analysis codes are available from corresponding author on reasonable request.

## Abbreviations

BMI: Body mass index
CET: Compulsive Exercise Test
DSM-5: Diagnostic and Statistical Manual for Mental Disorders, 5th edition
HRQoL: Health-related quality of life
PA: Physical activity
SDT: Self-determination theory
